# The dual role of the hexosamine biosynthetic pathway in cardiac physiology and pathophysiology

**DOI:** 10.3389/fendo.2022.984342

**Published:** 2022-10-24

**Authors:** Megan Cairns, Danzil Joseph, M. Faadiel Essop

**Affiliations:** ^1^ Centre for Cardio-Metabolic Research in Africa, Division of Medical Physiology, Faculty of Medicine and Health Sciences, Stellenbosch University, Cape Town, South Africa; ^2^ Centre for Cardio-Metabolic Research in Africa, Department of Physiological Sciences, Faculty of Science, Stellenbosch University, Stellenbosch, South Africa

**Keywords:** metabolism, heart, hexosamine biosynthetic pathway, oxidative stress, diabetes, cardiac hypertrophy, myocardial ischemia, heart failure

## Abstract

The heart is a highly metabolic organ with extensive energy demands and hence relies on numerous fuel substrates including fatty acids and glucose. However, oxidative stress is a natural by-product of metabolism that, in excess, can contribute towards DNA damage and poly-ADP-ribose polymerase activation. This activation inhibits key glycolytic enzymes, subsequently shunting glycolytic intermediates into non-oxidative glucose pathways such as the hexosamine biosynthetic pathway (HBP). In this review we provide evidence supporting the dual role of the HBP, i.e. playing a unique role in cardiac physiology and pathophysiology where acute upregulation confers cardioprotection while chronic activation contributes to the onset and progression of cardio-metabolic diseases such as diabetes, hypertrophy, ischemic heart disease, and heart failure. Thus although the HBP has emerged as a novel therapeutic target for such conditions, proposed interventions need to be applied in a context- and pathology-specific manner to avoid any potential drawbacks of relatively low cardiac HBP activity.

## Introduction

Next to the brain, the heart is the most energy-intensive organ in the body (1). Fuel substrate metabolism plays a key role to ensure optimal cardiac function. However, a fine balance exists between over- and under-utilization and a shift in either direction contributes towards cardiac dysfunction. Oxidation of a number of fuel substrates ensures a constant supply of ATP for cardiac contractility and ion gradient regulation (2). These energy substrates can be crudely divided into “*preferred*” (free fatty acids and glucose), or “*alternative*” (branched-chain amino acids [BCAAs] and ketones) which contribute 90% and 10%, respectively, towards cardiac ATP production (2). The dynamics between, and within, such substrate groups underlie the development of various pathologies ranging from diabetes to cardiac hypertrophy and heart failure (2, 3).

The switch from fatty acids to glucose as the main fuel substrate for energy production is commonly observed in heart failure, myocardial ischemia, cardiac hypertrophy, and increased cardiac workload (4–6). However, elevated glucose uptake and glycolysis rates in the hypertrophied heart do not necessarily correlate with increased pyruvate oxidation (7). This suggests that glucose enters glycolysis but that the rate of acetyl-CoA turnover in the Krebs cycle does not change – thus providing evidence for attenuated pyruvate dehydrogenase (PDH) activity. The delinking of glycolysis from mitochondrial oxidative phosphorylation promotes proton accumulation and a pro-oxidative milieu (8, 9).

Oxidative stress is a natural by-product of metabolism that can trigger intracellular damage ranging from DNA strand breaks to lipid and protein peroxidation (10). The damage mediated in this fashion leads to inhibition of glycolytic enzymes, including hexokinase, glyceraldehyde-3-phosphate dehydrogenase, and phosphofructokinase (PFK), through a combination of poly-ADP-ribose polymerase-dependent and independent mechanisms (11–16). This results in upstream glycolytic intermediate accumulation (11–15). Such intermediates are subsequently shunted into various non-oxidative glucose pathways (NOGPs) such as the polyol pathway, advanced glycation end-product (AGE) pathway, pentose phosphate pathway, and the hexosamine biosynthetic pathway (HBP). Although the NOGPs usually function at relatively low basal levels of activity, enhanced flux can occur with increased oxidative stress, or during hyperglycemic, diabetic, and hypertrophic conditions (13–15).

The links between elevated protein *O*-GlcNAcylation due to increased HBP flux and cardiac pathology are well established, particularly in the context of diabetes (17–19). The goal of this review is therefore not to reconsider the well-understood pathological mechanisms linked to diabetes due to chronic HBP activation. Rather, we aim to provide a contextualized understanding of the HBP in terms of cardiac function – from its acute and beneficial upregulation, to dysfunction induced by chronic *O*-GlcNAcylation across numerous cardiac pathologies. This includes a summary of available literature highlighting the significant findings of clinical trials, *in vivo, in vivo* transgenic, and *in vitro* studies regarding the impact of HBP regulation on the heart’s function.

## Hexosamine biosynthetic pathway and the heart

Inhibition of PFK increases intracellular fructose-6-phosphate which can be shunted into the HBP. Glutamine fructose-6-phosphate amidotransferase (GFAT) isoforms are location-specific with GFAT1 expressed in skeletal muscle and the heart, while GFAT2 is predominant in the heart, placenta, and spinal cord (20, 21). Nabeebaccus et al. (2021) recently found GFAT1 as the primary isoform in cardiomyocytes while GFAT2 is predominantly expressed in fibroblasts (22). Upon shunting into the HBP, GFAT1/2 converts fructose-6-phosphate into glucosamine-6-phosphate, which is metabolized to uridine diphosphate N-acetylglucosamine (UDP-GlcNAc) (23). *O*-GlcNAc transferase (OGT) is the primary enzyme responsible for catalyzing the reaction whereby *O*-GlcNAc binds to protein serine or threonine residues often in direct competition with phosphorylation (24). Binding to these sites may directly alter protein function, or indirectly alter alternative activation sites through steric or electrosteric actions (25). *O*-GlcNAcase (OGA) exerts opposing effects on OGT by hydrolyzing *O*-GlcNAc residues from proteins (24). The dynamics between OGT and OGA (in addition to GFAT1/2 activity) underlie the shift away from cardioprotection and towards cardiac pathology.

Detection of *O*-GlcNAc moieties on proteins can be crudely divided into two categories: overall and site-specific. The use of antibodies (particularly CTD110.6) designed to detect *O*-GlcNAcylated serine and threonine residues are most commonly utilized in Western blotting to determine the overall change in specific (*via* immunoprecipitation) or total protein modifications (26–29). The primary drawback of this method is the antibodies themselves as they lack specificity and may skew results based on protein abundance (30). Proteomic techniques such as β-Elimination followed by Michael addition (BEMAD) enable the identification of site-specific *O*-GlcNAcylation, thus allowing for greater understanding regarding the effects of *O*-GlcNAcylation in terms of protein function (31). However, this technique is most often applied at the peptide (versus full protein) level.

The HBP displays tissue and organ-specific expression profiles that fluctuate according to circadian rhythm, substrate availability, as well as age (32). Studies demonstrated the pivotal role of the HBP and downstream *O*-GlcNAcylation on pre- and post-natal cardiac development (33, 34). Complete OGT deficient animals display overt cardiac failure with severe cardiac fibrosis, apoptosis, and hypertrophy compared to wild-type littermates (33). Although *O*-GlcNAcylation is essential for normal cardiac development, Dupas et al. (2021) highlighted the time-dependent decrease in cardiac *O*-GlcNAc from birth (34). Such a decrease in *O*-GlcNAcylation suggests a shift away from non-oxidative glucose metabolism in the heart. Conversely, excessive protein *O*-GlcNAcylation is observed in multiple organs with increasing age in rat and mouse models, contributing to adverse clinical outcomes (35, 36). For example, young mice subjected to cardiac arrest and resuscitation displayed increased unfolded protein response pathways and post-translational modifications including *O*-GlcNAcylation, which corresponded with improved recovery not observed in aged animals (36, 37). The most accepted rationale behind this increase is the gradual shift into cellular senescence during the aging process and the subsequent decline of proteostasis (35, 36).

The influence of the HBP on cardiac function is gaining increasing attention and understanding (refer to **Tables 1**–**4** for a summary of currently available literature on the role of the HBP in the cardiovascular system). Here a comprehensive literature search was conducted using the search engines Scopus^®^ and PubMed, with keywords including “hexosamine biosynthetic pathway”, “*O*-GlcNAc”, and “heart” with the focus on original and clinical research article titles between the years 2000 and 2022. The most researched areas of *O*-GlcNAc pathology include the influence of altered glucose levels, ischemia-reperfusion injury (IR/I), hypertrophy, hypertension, and heart failure. These data reveal that the HBP is intricately involved in cardiac function and pathology and that *O*-GlcNAcylation affects various pathways and proteins (**Tables 1**–**4**). Such alterations may be protective or detrimental depending on the experimental and/or clinical contexts, and this often depends on the dynamic cycling, extent, and duration of protein *O*-GlcNAcylation.

**Table 1:** Summary of clinical studies (2000 – 2022) investigating the role of the HBP or *O*-GlcNAc in various pathological conditions.

Clinical studies						
Pathology	Average age	Study description	HBP	Heart function	Other significant findings	Ref
T1DM	n/a	This study aimed to assess the efficacy of HHC in T1DM patients with MI.	↑	↓	Reduced miRNA-24.	(38)
T2DM	55 ± 8 years	Endothelial cells were isolated from T2DM patients to elucidate the role of *O*-GlcNAc modification in endothelial dysfunction.	↑	n.d.	*O*-GlcNAc, Glc, and HbA1c are all directly proportional to each other.	(39)
T2DM	58 ± 4 years	Left ventricle tissue samples were collected from diabetic and non-diabetic patients.	↑	↓	↑ OGT and OGA in the myocardium	(40)
PAH	53.7 ± 16.0 years	Endomyocardial biopsy samples were collected from PAH patients to investigate the relationship between *O*-GlcNAcylation and RV function.	↑	↓	Increased HbA1c.	(41)
IPAH	38.3 ± 16.0 years	Human lung tissues from IPAH patient donor lung explants were used to determine the relationship between altered glucose metabolism and smooth muscle cell proliferation.	↑	n.d.		(42)
IPAH	n/a	Human lung tissues from IPAH patient donor lung explants were utilized to investigate the link between *O*-GlcNAc and eNOS function.	↑	n.d.		(43)
Aortic stenosis	63.9 ± 4.8 years	Left ventricular apical myocardial biopsies were collected during elective aortic valve replacement, and from a non-ischemic area during coronary artery bypass operation.	↑	↓		(44)
Severe aortic stenosis	70 ± 10 years	Patients undergoing aortic valve replacement were treated with HNC or insulin to assess the efficacy of HNC as a treatment.	–	–	↓ time-weighted mean [Glc] with no evidence of increased glycolytic pyruvate oxidation.	(45)
T2DM + HF	57 ± 4.9 years	Left ventricular biopsies were obtained from diabetic patients with end-stage HF (EF < 20%) to assess ketone gene expression.	n.d.	↓	↓ ketone utilization	(46)
T2DM + HF	42 – 60 years	Failing hearts obtained during orthotopic heart transplantation.	↑	↓	↑ CaMKII *O*-GlcNAcylation	(47)
HF	54 ± 4 years	The role of *O*-GlcNAcylation in cardiac hypertrophy was assessed in HF patient (EF < 25%) samples.	↑	↓	–	(48)

Search results were obtained using Scopus^®^ and PubMed; key words including “hexosamine biosynthetic pathway”, “*O*-GlcNAc”, and “heart” with focus on original, clinical research article titles. The average age is expressed as Mean +/- standard deviation. Only studies that assess either the HBP or *O*-GlcNAc from human samples were included. CHF, congestive heart failure; DM, diabetes mellitus; EF, ejection fraction; Glc, glucose; HBP, hexosamine biosynthetic pathway; HF, heart failure; HHC, hyperinsulinemic-hypoglycemic clamp; IPAH, idiopathic pulmonary artery hypertension; n/a, not available; PAH, pulmonary artery hypertension; PCR, polymerase chain reaction; T1DM, type 1 diabetes mellitus; T2DM, type 2 diabetes mellitus; CaMKII, calcium-calmodulin dependent protein kinase II. "↑ " = increase "↓" = decrease "-" = no change n.d. = no data

**Table 2:** Summary of available literature (2000 – 2022) utilizing non-transgenic animal models to investigate the role of the HBP or *O*-GlcNAc in various pathologies.

*in vivo* (non-transgenic animal models)					
Animal model	Study description	HBP	Heart function	Downstream implications	Ref
Obesity/MetS	Influence of *O*-GlcNAc protein modifications cardiac dysfunction.	↑	↓	ns ∆ ER stress and blood Glc; ↑ OGT/OGA ratio; ↓ FFA metabolism; ↑ ketones; protein *O*-GlcNAcylation more dependent on OGT and OGA dynamics than HBP activity; ↑ REST *O*-GlcNAcylation; ↓ HDAC2 and α-actin	(49–53)
Diabetes/chronic hyperglycemia					
- Sugar-sweetened beverages	Effects of chronic sugar-sweetened beverages on cardiac function.	↑	–	↑ HbA1c; ↑ weight gain (no change in insulin resistance), ↑ *O*-GlcNAc (3 months)	(54)
- Induced T1DM and T2DM	Investigating the role of *O*-GlcNAc protein modifications of several processes including cell death pathways, nutrient sensing, DNA repair, mitochondrial function, calcium handling as well as excitation and contractility of cardiomyocytes.	↑	↓	↑ Ca^2+^ sensitivity/capacity; ↑ apoptosis/autophagy ratio; ↑ interstitial fibrosis; ↑ mitochondrial dysfunction; ↓ sodium channel function; ↑ ox. Stress/inflammation; ↑ DNA damage; hypertrophy; ↓ ketone utilization/production ratio; ↑ ERK1/2 and cyclin D2 expression; ↑ CaMKII *O*-GlcNAcylation and autophosphorylation; ↑ p-CREB; ↓ p-cTnI; ↑ Nkx2.5 *O*-GlcNAcylation	(26, 29, 46, 47, 55–72)
Hypertension	Evaluating the influence of hypertension in the absence of diabetes on HBP flux as well as RV and endothelial function.	↓/↑	↑/↓	Monocrotaline: ↑ p-AMPK → ↓ HBP and ↑ heart function. DOCA-salt: ↑ HBP → ↓ eNOS → ↓ vasorelaxation	(41, 73)
IR/I:					
- Intermittent hypoxia	The impact of *O*-GlcNAcylation on pathological cardiac remodeling and dysfunction in intermittent hypoxia.	↑	n.d.	↑ apoptosis and inflammation; ↑ p- ERK1/2 and p38-MAPK; ↑ BP	(74)
- Hypoxia vs reperfusion	Variation in ischemic region *O*-GlcNAc expression.	↑/↓	n.d.	Hypoxia → ↓ ischemic *O*-GlcNAc expression; reperfusion *O*-GlcNAc peaks at 1hr post reperfusion	(75)
- Hypoxic acclimation	The impact of hypoxic acclimation on IR/I and the mechanisms thereof.	↑	↑	↓ infarct size; ↓ ox. stress; ↑ *O*-GlcNAcylation → ↑ NADPH/NADP^+^ and GSH/GSSG couples	(76)
- Preconditioning	The cardioprotective capacity of IPC under diabetic and non-diabetic conditions after IR.	↑	↑	↑ MGU; ↓ infarct size	(77–79)
- Hypoglycemia	Impact of hypoglycemia in diabetic heart IR/I, and the efficacy of IPC.	↑	n.d.	↑ MGU	(80)
- Acute Hyperglycemia	The role of *O*-GlcNAc in IR/I was observed during acute hyperglycemia.	↑	↓	↑ apoptosis; ↑ IR/I	(81–83)
- Chronic hyperglycemia	Investigating the underlying mechanisms of *O*-GlcNAc PTMs in IR/I.	↑	↓	↑ HbA1c; ↓ antioxidant capacity; ↑ apoptosis	(38, 54, 81, 82, 84)
Cardiac hypertrophy	Influence of pressure overload on the HBP.	↑	↓	HBP ∝ hypertrophy	(44, 85–87)
Septic shock	Effects of *O*-GlcNAc stimulation using two models of septic shock (LPS and CLP).	↑	↑	normalized SERCA2α	(88)
Trauma-hemorrhage	Evaluating the effects of glucosamine on inflammatory signaling.	↑	↑	↓ NF-κB nuclear translocation; ↓ ALT, AST, and LDH → improved prognosis	(89–93)
Heart failure					
- Desmin	Characterizing the interplay between desmin phosphorylation and *O*-GlcNAcylation.	↓	n.d.	↑ p-desmin/desmin *O*-GlcNAc ratio; ns ∆ in total desmin	(94)
- TnT	Effects of TnT phosphorylation and *O*-GlcNAcylation.	↑	↓	↑ TnT *O*-GlcNAc/phosphorylation ratio; ↑ OGT/OGA activity	(95)
- miRNA-539	Role of miRNA-539 in heart failure.	↑	↓	↑ miRNA-539 → ↓ OGA expression	(96)

Although the table below contains a large proportion of available literature, not every original research paper could be included. Search results were obtained using Scopus^®^ and PubMed; keywords including “hexosamine biosynthetic pathway”, “*O*-GlcNAc”, and “heart” with a focus on original research article titles. A handful of studies could not be obtained due to limited access and were thus excluded. ex vivo studies that did not include any in vivo experimentation were also excluded from the following table. Articles investigating the HBP on endothelial function were not included. T1DM, type 1 diabetes mellitus; T2DM, type 2 diabetes mellitus; HBP, hexosamine biosynthetic pathway; IR, ischemia-reperfusion; IR/I, ischemia-reperfusion injury; DNA, deoxyribonucleic acid; MGU, myocardial glucose uptake; HbA1c, glycosylated hemoglobin; ER, endoplasmic reticulum; mPTP, mitochondrial permeability transition pore; OGA, *O*-GlcNAcase; OGT, *O*-GlcNAc transferase; ERK - extracellular signal-regulated kinases; MAPK, mitogen-activated protein kinase; BP, blood pressure; PTM, post-translational modifications; PAB, pulmonary aortic banding; p-AMPK, phosphorylated 5' adenosine-monophosphate-activated protein kinase; MCT, monocrotaline; RV, right ventricle; LPS, lipopolysaccharide; CLP, cecal ligation puncture; IPC, ischemic preconditioning; MetS, metabolic syndrome; PFK1, phosphofructokinase 1; ALT, alanine transaminase; AST, aspartate transaminase; LDH, lactate dehydrogenase; REST, repressor element 1-silencing transcription factor; HDAC2, histone deacetylase 2; ns, non-significant; ↑, increase; ↓, decrease; -, no change; n.d., no data.

**Table 3:** Summary of available literature (2000 – 2022) utilizing transgenic rat and mouse models to investigate the role of the HBP or *O*-GlcNAc in various pathologies.

*in vivo* (transgenic)					
Research focus	Study Description	HBP	Heart function	Other significant results	Ref
Metabolic reprogramming					
- Decorin^KO^	Role of the extracellular matrix in cardiac fasting metabolism.	↑	**↑**	↓ autophagy	(97)
- Ivabradine and metoprolol	Comparing treatment efficacy in dyslipidemia.	↑	**↓**	↑ p-Akt	(98)
- G6PDH deficiency	Effects of G6PDH in response to obesogenic or high-fructose diets.	↓	**↑**	↓ G6PDH → cardioprotection under diet-induced metabolic stress.	(99)
Diabetic cardiomyopathy					
- Histone modifications	Investigating the balance between adaptive and maladaptive site-specific *O*-GlcNAc modifications of HDAC4 and its regulators.	↑	**↑**	*O*-GlcNAcylation at S642 inverse relationship with CaMKII binding at S632	(100)
- Lipotoxicity	Independent assessment of gluco- and lipo-toxicity in a Seipine^KO^ T2DM model of diabetic cardiomyopathy.	↑	**↓**	↓ p- phospholamban, ↓ O_2_ consumption; ↑ FOXO1 activity	(101)
- Atrial fibrillation	The interplay between *O*-GlcNAcylation and ROS-mediated oxidative phosphorylation of CaMKII under diabetic conditions.	↑	**↓**	ROS and *O*-GlcNAc promote atrial fibrillation through CaMKII-dependent and independent mechanisms, respectively.	(102)
- AdOGA	Influence of p53 *O*-GlcNAcylation cardiovascular functionality.	**↓**	**↑**	↑ OGA → ↓ p53	(62)
- Substrate utilization	The shift in substrate preference observed in the failing heart through inducible GLUT4 and OGA^-/-^ mouse models.	↑	n.d.	↑ Glc and *O*-GlcNAc → ↓ ketone synthesis	(46)
- Drosophila model	A combination of diet, genetics, and physiology to establish a model of chronic high sugar-induced heart disease.	↑	**↓**	↑ fibrosis and fat deposition; ↓ insulin signaling; ↓ life expectancy; HBP ∝ cardiac dysfunction	(103)
- rAAV6-OGA	Investigating the role of OGA and OGT in the presence of diabetes.	**↓**	**↑**	Preserved PI3K-Akt signaling	(40)
Intermittent hypoxia	Investigating the effect of IH on HBP activity through OGT overexpression.	**↑**	n.d.	↓ cardiac remodeling; ↑ GSK-3b and NF-kB p65 *O*-GlcNAcylation; ↓ rate of ox. stress and apoptosis	(104)
IR/I					
- OGT^KO^	Contribution of OGA in a post-hypoxic setting.	↓	↓	↓ VDAC *O*-GlcNAc → ↑ mPTP	(105)
- E2f1^KO^	The effects of an E2f1 on *O*-GlcNAcylation and heart function.	**-**	**↑**	↑ heart size	(106)
- BCAA	Investigating the molecular mechanisms linking elevated BCAAs on cardiac glucose metabolism and function.	↓	**↓**	↓ PDH, ↑ FFA oxidation/Glc oxidation, ↑ IR/I susceptibility	(107)
- miR-24	The role of miR-24 overexpression on myocardial function in diabetic and non-diabetic conditions.	↓	n.d.	↓ infarct size; ↓ fasting plasma glucose and insulin	(38)
- Xbp1^KO^	Investigating the influence of Xbp1 on HBP regulation.	**↓**	**↓**	Xbp1 is required for HBP activation	(108)
Hemodynamic stress	The role of Nox4 in altered cardiac energy metabolism, remodeling, and function.	↑	n.d.	↑ NOX4 → ↑ global *O*-GlcNAc; ↓ glycolysis; ↑ palmitate oxidation	(109)
Hypertrophy					
- OGT^KO^	Investigating the influence of reduced HBP flux on heart function.	↓	**↓**	↓ p-phospholamban and p-cardiac troponin I; ↑ TGF-β and ↓ GATA-4; ↑ PGC-1α	(48, 110, 111)
- AdGFAT	Insight into the mechanisms underlying increased *O*-GlcNAc PTMS observed in cardiac hypertrophy.	↑	**↓**	↑ hypertrophy; ↑ relative heart weight; ↑ fibrosis; ↑ mTOR	(85)
- AMPK^KO^	Elucidating the underlying mechanisms of cardiac hypertrophy through an AMPK^KO^ model.	**↑**	n.d.	↑ ANGII increases *O*-GlcNAc regardless of AMPK; ↑ AMPK → ↓ *O*-GlcNAc	(112)
- Myc^KO^	Investigating the dynamics between Myc, substrate oxidation, and cardiac function in hypertrophy.	**↑**	**-**	↓ ketone and FFA contribution to Krebs cycle	(113)
- LXRα	The role of LXRα in cardiac hypertrophic pathogenesis.	**↑**	n.d.	↑ MGU → ↑ GATA4 and Mef2c *O*-GlcNAcylation; ↑ ANP	(114)
Heart failure					
- OGA/OGT	Relationship dynamic between *O*-GlcNAc and heart failure.	**↑/**↓	**↓**	*O*-GlcNAc ∝ autophagy and impaired mitochondrial complex I activity; ↓ *O*-GlcNAc exacerbates dysfunction in IR/I; ↓ OGA → ↑ early infarct damage	(115–118)
- Hdac4-NT	Therapeutic effects of the proteolytic fragment.	↓	↑	Hdac4-NT inhibits HBP activity through NR4A1	(119)
- TnT	Effects of TnT phosphorylation and *O*-GlcNAcylation.	↑	**↓**	Inverse relationship between TnT *O*-GlcNAc and phosphorylation	(95)

Search results were obtained using Scopus^®^ and PubMed; keywords including “hexosamine biosynthetic pathway”, “*O*-GlcNAc”, and “heart” with a focus on original research article titles. A handful of studies could not be obtained due to limited access and were thus excluded. p-Akt, phosphorylated protein kinase B; ROS, reactive oxygen species; FOXO1, forkhead box protein O1; CaMKII, Ca^2+^/calmodulin protein-dependent kinase II; PDH, pyruvate dehydrogenase; FFA, free fatty acid; HBP, hexosamine biosynthetic pathway; ANGII, angiotensin II; AMPK, 5' adenosine-monophosphate-activated protein kinase; GFAT, glutamine fructose-6-phosphate amidotransferase; TGF-β, transforming growth factor-beta; GATA-4, GATA Binding Protein 4; mTOR, mammalian target of rapamycin; XBP1, X-box binding protein 1; VDAC - voltage-dependent anion channel; mPTP, mitochondrial permeability transition pore; GATA4, GATA Binding Protein 4; Mef2c, myocyte-specific enhancer factor 2C; ANP, atrial natriuretic peptide; ↑, increase; ↓, decrease; -, no change; n.d., no data.

**Table 4:** Summary of available literature (2000 – 2022) utilizing *in vitro* models to investigate the underlying mechanisms between the HBP or *O*-GlcNAc in various pathologies.

*in vitro*						
Research focus	Experimental Overview	HBP	Ox. stress	Apoptosis	Downstream effects	Ref
PTMs:						
- Histone	Effects of Glc and *O*-GlcNAcylation on HDAC4.	↑	n.d.	n.d.	↑ HDAC4 *O*-GlcNAcylation	(100)
- Sarcomere	Desmin phosphorylation and *O*-GlcNAcylation dynamics.	↑/↓	n.d.	n.d.	No ∆ IP desmin/total desmin	(94)
- Contractile proteins	Co-localization of *O*-GlcNAc and proteins.	↑	n.d.	n.d.	↑ ZASP *O*-GlcNAcylation	(120)
- αB-crystallin	Influence of *O*-GlcNAcylation on translocation and function.	↑	n.d.	↓	↑ αB-crystallin translocation	(121)
- Phospholamban	Relationship between phospholamban, SERCA2a, and *O*-GlcNAc.	↑	n.d.	n.d.	↑ phospholamban *O*-GlcNAcylation → ↓ p-phospholamban → ↓ cardiac function	(122)
- β1AR	Effects of OGT on β1AR function.	↑	n.d.	↓	↑ β1AR *O*-GlcNAcylation → ↓ cAMP and p-phospholamban	(123)
Mitochondrial						
- BCAAs	Effects of BCAAs and *O*-GlcNAc cycling.	↓	n.d.	n.d.	↓ PDH activity; ↓ mitochondrial membrane potential	(65, 107)
- OGA^KO^	Impact of *O*−GlcNAc on mitochondria.	↑	n.d.	n.d.	↑ fission; ↓ mass; ↓ ETC activity	(124)
Inflammation	Influence of *O*-GlcNAc on oxidative stress with a focus on iNOS expression, A20 PTM, as well as the effects of glucosamine on NF-κB signaling.	↑	↓	n.d.	↓ pro-inflammatory cytokines; *O*-GlcNAc levels inversely proportional to inflammatory stimuli response	(34, 89, 90)
Diabetic arrhythmia	Effects of Nav1.5 and CaMKII *O*-GlcNAcylation on the progression of diabetic cardiomyopathy to arrhythmias.	↑	n.d.	n.d.	↓ sodium channel function; CaMKII serine^280^ *O*-GlcNAc not required for activation but serine^279^ *O*-GlcNAc mediates activity	(57, 58, 125)
Glucose levels						
- Glc deprivation	The effect of Glc deprivation on *O*-GlcNAc levels.	↑	n.d.	n.d.	↓ CaMKII → ↓ *O*-GlcNAc	(126)
- Acute hyperglycemia	Role of *O*-GlcNAc post-translational protein modifications on cardioprotective enzyme activity as well as cardiac Glc uptake, apoptosis, and ion channel polarization.	↑	↑	↑	↑ IR/I; ↓ insulin-mediated Glc uptake; ↑ aldehydes; ↑ I_K1_ and I_to_ recovery *via* CaMKIIδ-S280 *O*-GlcNAcylation; ↓I_to_ amplitude; ↓ autophagic signaling	(28, 29, 81, 82, 127, 128)
- Chronic hyperglycemia	Investigating the influence of *O*-GlcNAc in serval processes including mitochondrial dysfunction, ion channel polarization, DNA repair machinery, nutrient sensing, fibrosis, as well as antioxidant defense and apoptotic systems.	↑	↑	↑	↑ IR/I; ↓ p-AMPK; ↑ aldehydes; ↓ K^+^ channel expression and function; ↓ eNOS activation; ↓ autophagic flux, ↑ collagen, ↓ mitochondrial respiration, and ↑ fragmentation; ↓ Ca^2+^ cycling; ↑ SGLT1 Glc transport → ↑ NOX2 activation	(27, 39, 40, 50, 55, 60, 68, 81, 82, 127, 129–134)
IR/I:						
- Hypoxia	Investigating the effect of IH on HBP activity and mitochondrial function.	↑	↓	↓	↓ GSK-3β and NF-κB activity; ↑ OGA → ↑ ROS but no change to Ca^2+^ overload; ↑ Bcl-2	(75, 104, 105, 135)
- Xbp1	Investigating the regulatory actions of Xbp1 on HBP flux.	↑	n.d.	↓	Xbp1 ∝ GFAT1 expression; ↑ ER stress	(108)
- miRNA-24	The role of miR-24 overexpression in diabetic conditions.	↓	n.d.	↓	↓ ATG4a	(38)
- OGA	Contribution of OGA on cardioprotection.	↓	n.d.	↑	↓ mitochondrial Ψ_M_ recovery	(136)
- ER stress	Role of *O*-GlcNAc in ER stress signaling.	↑		↓	↓ CHOP activation	(137)
PAH	Role of *O*-GlcNAc, eNOS, and cellular proliferation.	↑	↑	n.d.	↑ cell proliferation	(41–43)
Hypertrophy						
- ↑ HBP	Molecular mechanisms linking the HBP to cardiac hypertrophy through GFAT overexpression, high Glc, phenylephrine, NOX4, and STIM1 models.	↑	n.d.	**-**	↑ mTOR; ↑ cell cross-section area; ↑ FFA ratio; ↓ hypertrophic response to ANG and PE; ↑ ERK1/2 and cyclin D2 expression; NFAT *O*-GlcNAc required for hypertrophy; ↑ STIM1 *O*-GlcNAc → ↓ SOCE; ↑ p-CREB	(57, 58, 69, 85, 87, 109, 138)
- ↑ OGA	The role of *O*-GlcNAc in cardiac hypertrophy signaling.	↓	n.d.	n.d.	↑ TGF-β and ↑ PGC-1a; ↓ GATA-4; ↓ ANP	(48, 111)
- AMPK	The dynamic between AMPK and *O*-GlcNAcylation.	↓/↑	n.d.	n.d.	↑ AMPK → ↓ *O*-GlcNAc → ↓ hypertrophy	(112)
Heart failure						
- miRNA-423-5p	The relationships between miR-423-5p and downstream targets such as OGT, and apoptosis.	↓	n.d.	↑	↑ AMPK/p-AMPK ratio; ↑ 26S proteasome activity	(139, 140)
- miRNA-539	The relationship between mRNA-539 and OGA.	↑	n.d.	n.d.	Hypoxia-reoxygenation → ↑ miRNA-539 → ↓ OGA	(96)
- siOGT	The relationship dynamic between *O*-GlcNAc and nutrient-sensing observed in heart failure.	↓	n.d.	n.d.	OGT required for autophagy	(116)
- HDAC4^KO^	Mechanisms linking heart failure to HBP activity.	↑	n.d.	n.d.	↑ Nr4a1 → ↑ STIM1 *O*-GlcNAcylation → ↓exercise capacity	(119)

Although the table below contains a large proportion of available literature, not every original research paper could be included. Articles investigating the HBP on endothelial function were not included. Search results were obtained using Scopus^®^ and PubMed; keywords including “hexosamine biosynthetic pathway”, “*O*-GlcNAc”, and “heart” with a focus on original research article titles. A handful of studies could not be obtained due to limited access and were thus excluded. PTM, post-translational modifications; HBP, hexosamine biosynthetic pathway; IP, immunoprecipitated; Glc, glucose; IR/I, ischemia-reperfusion injury; CaMKII, calcium-calmodulin protein-dependent kinase II; GIK, glucose-insulin-potassium; SCN, suprachiasmatic nucleus; ER, endoplasmic reticulum; PFK2, phosphofructokinase 2; β1AR, β1-adrenoceptor; NFAT, nuclear factor of activated T-cells; Phe, phenylephrine; NOX4, NADPH oxidase-4; p-CREB, phosphorylated cAMP response element-binding protein; MGU, myocardial glucose uptake; NOX2, NADPH oxidase 2; SGLT1, sodium/glucose cotransporter; PAH, pulmonary artery hypertension; ETC, electron transport chain; A20, tumor necrosis factor α-induced protein 3; ZASP, Z-band alternatively spliced PDZ motif protein; ↑, increase; ↓, decrease; -, no change; n.d., no data.

The following sections aim to highlight the potential benefits and harms of altered HBP and *O*-GlcNAcylation in both pre-clinical and clinical studies (**Tables 1**–**4**). The benefits of acute HBP flux on the heart will also be discussed, followed by the effects of chronic activation.

### HBP flux: Benefits of acute upregulation

While the majority of literature highlights the detrimental effects of excessive HBP activation, increased flux can be beneficial within an acute context. *O*-GlcNAcylation appears to promote cellular survival, for e.g. exposure to bacterial lipopolysaccharide or trauma events increases HBP activity. Here, lipopolysaccharide increases *O*-GlcNAcylation of calcium transporters (e.g. L-type subunits of the sarcoplasmic reticulum Ca^2+^-ATPases (LTCC)) and enhances cardiac contractility (88, 141), whereas attenuated inflammation and increased *O*-GlcNAcylation occur as a result of trauma-hemorrhage events (89–93). Moreover, increased HBP activity is indirectly proportional to both inflammatory and oxidative markers in an acute context, with the opposite observed in a chronic setting (142–144). *O*-GlcNAcylation also influences cardiomyocyte apoptosis. αB-crystallin is an intracellular chaperone that prevents the toxic accumulation of misfolded proteins and inhibits apoptosis when active. While the majority of post-translational modifications (PTMs) decrease αB-crystallin’s function as a molecular chaperone, *O*-GlcNAcylation on threonine^170^ attenuates cardiomyocyte apoptosis through increased αB-crystallin activity and translocation (121). While this is beneficial in an acute context, chronic HBP activation is associated with increased apoptosis and diminished autophagy (17, 74, 145, 146). Findings from our laboratory showed higher apoptosis under simulated hyperglycemic conditions that were linked to *O*-GlcNAcylation of the pro-apoptotic protein Bad, with a concomitant decrease in its phosphorylation (27). This further highlights the context-specific, dual nature of HBP activity in the heart.

With diminished autophagic and apoptotic signaling, cardiac cells should face increased hypertrophic stimuli. In contrast, enhanced *O*-GlcNAcylation of histone deacetylase 4 (HDAC4) serine^642^ elevates its activity and hinders gene transcription, ultimately lowering transforming growth factor-beta (TGF-β) myoblast differentiation and hypertrophy (100). While this may appear to prevent cardiomyocyte differentiation and hypertrophy in an acute context, *O*-GlcNAc also binds to the theonine^63^ residue of mothers against decapentaplegic homolog 4 (SMAD4), stabilizing it and preventing its binding to glycogen synthase kinase 3β, and ultimately augmenting downstream TGF-β signaling (147). Thus a combination of SMAD-independent (i.e. mammalian target of rapamycin) and SMAD-dependent signaling may compensate for reduced TGF-β/glycogen synthase kinase 3β pathway activity in the long term and thereby contribute to cardiac hypertrophy.

Acute HBP upregulation can therefore confer cardioprotection against hypertrophy and apoptosis, as well as improved functionality. Although various proteins require *O*-GlcNAcylation for activation or repression under normal physiological conditions, constant *O*-GlcNAcylation can potentiate protein dysfunction and cardiac pathology, for example in the context of ischemia-reperfusion.

### Dual role of *O*-GlcNAcylation in ischemia-reperfusion

**Figure 1:**
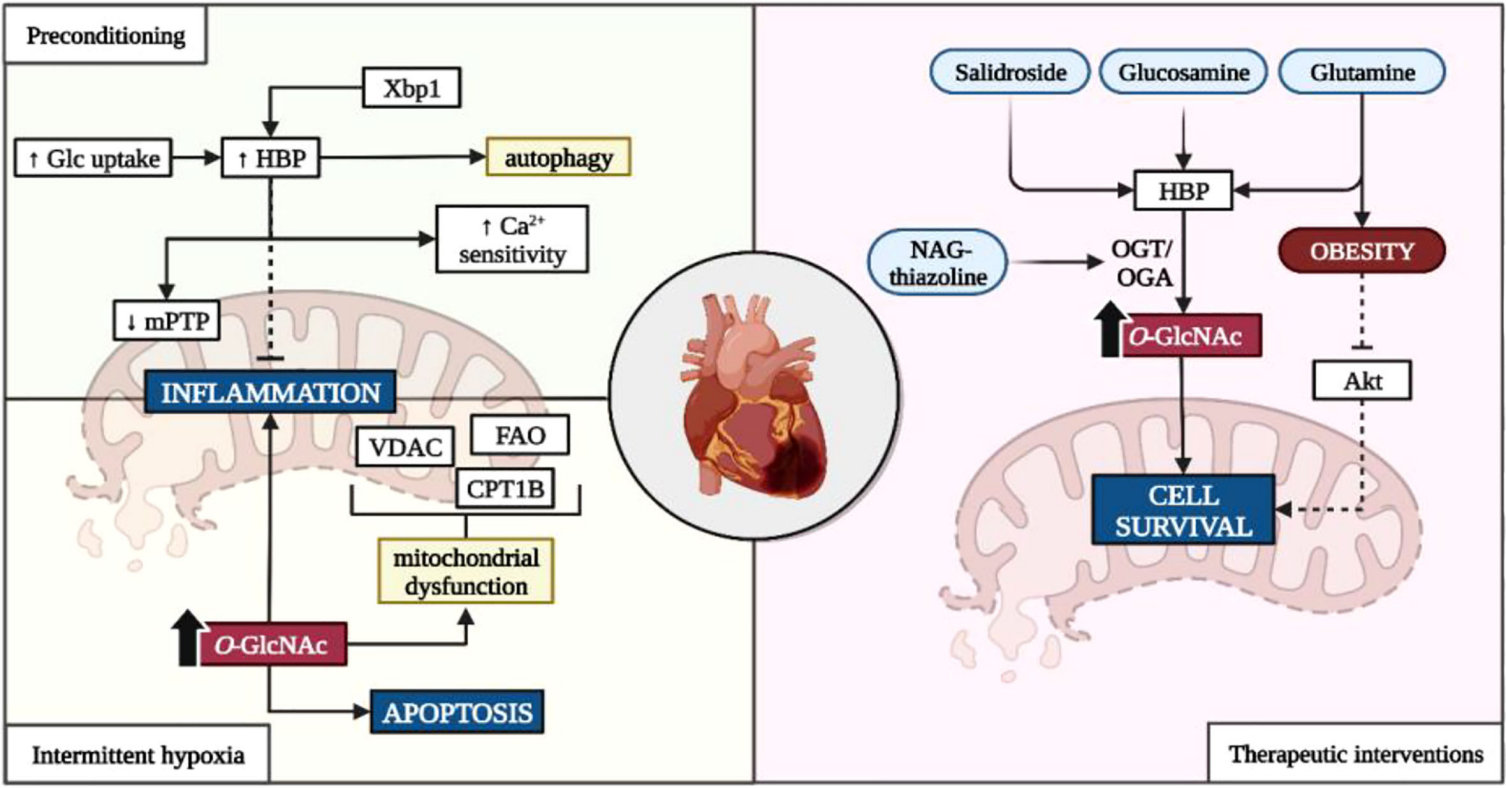
Influence of the HBP on ischemia-reperfusion injury. Glc, glucose; HBP, hexosamine biosynthetic pathway; Xbp1, X-box–binding protein-1; Ca^2+^, calcium; VDAC, voltage-dependent anion channels; CD36, cluster of differentiation 36; CPT1, carnitine palmitoyltransferase-1B; OGT, *O*-GlcNAc transferase; OGA, *O*-GlcNAcase; NAG, 1,2 dideoxy-2’-methyl-α-D-glycopyranoso-[2,1-d]-∆2’-thiazoline. *Created with BioRender.com*.

Depending on the duration and frequency of the ischemic event, *O*-GlcNAcylation is either protective or detrimental to cardiac function and health (**Figure 1**). For example, increased HBP flux is often observed during ischemic preconditioning, intermittent hypoxia, and acute IR/I episodes where it confers cardioprotection (77, 78). Here, increased myocardial glucose uptake elevates myocardial *O*-GlcNAc levels during ischemic preconditioning and improves mitochondrial function through increased calcium sensitivity and reduced mitochondrial transition pore opening (mPTP) (148). Subsequently, this will minimize myocardial infarct damage following reperfusion under experimentally induced diabetic and non-diabetic conditions (77, 78, 148, 149). Acute upregulation of X-box binding protein 1 (Xbp1) due to endoplasmic reticulum stress during IR/I increases HBP flux and confers cardio-protection by increasing autophagy of misfolded proteins (108).

Exposure to intermittent hypoxia also upregulates the HBP which can either suppress inflammatory and hypertrophic genes (i.e. nuclear factor kappa-light-chain-enhancer of activated B cells (NFκB) and nuclear factor of activated T cells (NFAT)), or promote apoptotic and inflammatory signaling depending on the duration of the ischemic event (74, 104). Consistent *O*-GlcNAcylation of mitochondrial proteins such as voltage-dependent anion channels (VDAC), carnitine-palmitate transferase 1B (CPT1B), and fatty acid oxidase (FAO) contributes to attenuated fatty acid β-oxidation, impaired calcium handling, increased mitochondrial fragmentation, and overall mitochondrial dysfunction (55, 56, 150). Moreover, elevated *O*-GlcNAcylation further hinders cardiac function through inflammatory and apoptotic signaling, and promotes hypertension (38, 54, 81, 82). This is further highlighted in diabetic studies, where chronically elevated glucose conditions exacerbate ischemic injury. Whilst the exact mechanisms remain unclear, Liu et al. (2017) suggested that the reduction in mitochondrial aldehyde dehydrogenase 2 (ALD2) activity due to its *O*-GlcNAcylation, impedes its protective effects and augments myocardial injury (82).

Although downregulating HBP activity should abate the negative consequences of chronic HBP activation, this is not always the case. Repression of OGT or OGA upregulation are primary intervention targets that are influenced by E2F transcription factor 1 (E2f1). E2f1 promotes entry into the S-phase of the cell cycle and alters metabolism by increasing pyruvate dehydrogenase kinase 4 activity which delinks glycolysis from mitochondrial oxidative phosphorylation (151). Increased E2f1 activity also potentiates phosphatase and tensin homolog-induced kinase 1 (PINK1) downregulation through miRNA-421 transcription, resulting in mitochondrial fragmentation and attenuated cardiomyocyte energetics (152). However, some like Dassanayaka et al. (2019) demonstrated that the lowering of E2f1 activity improved myocardial injury and cardiac remodeling without any effects on the HBP (106). This suggests that alternative NOGPs may utilize the accumulating glycolytic intermediates upstream of PDH, as opposed to the HBP.

Various interventions attempted to harness the potential benefits of an upregulated HBP in the context of ischemia (refer **Figure 1**). The most frequently reported compounds include salidroside, glucosamine, and glutamine as well as OGA inhibitors such as NAG-thiazoline due to their anti-hypoxia and pro-survival properties (135, 153–156). These treatments increase flux into the HBP through upregulated myocardial glucose uptake or as substrate intermediates within the HBP, as well as facilitating protein *O*-GlcNAcylation through OGT/OGA dynamics (135, 153–156). Although the exact mechanisms have not been fully elucidated, evidence suggests that protein *O*-GlcNAcylation influences Bcl-2 translocation (an intrinsic part of cell-death machinery) and cytoplasmic calcium concentration which mitigate injury following reperfusion (135, 155). However, the protective effects of glucosamine are diminished in obesity where increased HBP activity prevents insulin-dependent protein kinase B (Akt) signaling, thus hindering cell survival post-infarction (84).

Clinical and pre-clinical studies demonstrate the dual nature of the HBP during ischemic events. Increased HBP activity during mild ischemic or ischemic preconditioning protects the heart against ischemic injury. However, these effects do not extend to prolonged ischemic conditions that are associated with chronic HBP upregulation. Under such conditions, the HBP appears to be detrimental to cardiac health and function by disrupting normal cardiac metabolism and mitochondrial bioenergetics. Consistent *O*-GlcNAcylation of key proteins involved in these processes potentiate cardiac dysfunction and contribute towards increased IR/I.

### Chronic HBP upregulation: Development of hypertension and cardiac hypertrophy

Clinical studies on idiopathic pulmonary artery hypertension observed increased HBP activity and circulating HbA1c levels with decreased heart function (41–43). *In vivo* experiments suggest increased HBP activation contributes to the lowered cardiac function observed in the clinical context (**Tables 2**, **3**). Although the mechanisms underpinning such observations vary, elevated *O*-GlcNAcylation regulates adenosine monophosphate-activated protein kinase (AMPK) and endothelial nitric oxide synthase activity (eNOS) (43, 157). Here, *O*-GlcNAcylation of AMPKα and γ subunits increases its activity, which in turn results in AMPK phosphorylation of GFAT1/2 and OGT (threonine^144^), and creates a negative feedback loop in terms of HBP activity (112, 157). This bi-directional relationship improves heart function and limits cardiac hypertrophy in the event of increased AMPK activity and attenuated HBP flux (41, 73, 112). Interestingly, angiotensin II increases *O*-GlcNAcylation in the presence of active AMPK although the exact mechanism remains unclear (112).

Site-specific PTMs regulate eNOS synthase activity function, i.e. activated by serine^1177,615,633^ phosphorylation and inhibited by threonine^495^ phosphorylation (158). *O*-GlcNAc targets serine^1177 and 615^ without influencing other phosphorylation sites and lowering eNOS activity (43, 158). As a result, less nitric oxide is produced to subsequently lower vasodilatory capabilities and contributes to decreased vascular tone. Increased HBP flux promotes intracellular oxidative stress and cellular proliferation, further augmenting vascular dysfunction (41–43, 73). Nabeebaccus et al. (2017) observed that higher NAD(P)H oxidase 4 (NOX4) expression levels increased global *O*-GlcNAcylation through an activating transcription factor 4 (ATF4)-mediated increase in GFAT1 expression while shifting cardiac metabolism towards free fatty acid (FFA) β-oxidation *versus* glycolysis (109). Moreover, protein and mRNA analyses revealed a marked increase in cluster of differentiation 36 (CD36) *O*-GlcNAcylation (a major transporter of FFAs into the cytosol) which increases its activity and FFA influx into cardiomyocytes (109). Increased FFA β-oxidation can attenuate glycolysis *via* the Randle cycle enhancing HBP flux through upstream glycolytic substrate accumulation, and/or by higher GFAT1/2 expression. Although increased FFA β-oxidation is less efficient than glucose oxidative phosphorylation, mitochondrial bioenergetics and cardiac function were preserved in this case (109). This suggests that increased FFA β-oxidation may not be as detrimental to cardiac function in a hypertensive context, but that this may not hold when combined with other pathological complications such as ischemic events (reviewed by (159)).

Although cardiac hypertrophy is commonly observed under persistent hypertension, its development under diabetic and non-diabetic conditions remains a contentious topic. For example, under non-diabetic conditions, some indicated that the attenuated cardiac function observed in the hypertrophied heart (with increased HBP flux) is directly proportional to mammalian target of rapamycin (mTOR) activity (85). This resulted in increased protein synthesis and cellular growth, ultimately potentiating cardiac hypertrophy and fibrosis (85). However, others who characterized HBP flux in an *ex vivo* murine heart concluded that neither acute changes in glucose availability nor cardiac workload influenced its flux in a healthy heart (160). *In vivo* diabetic studies concluded that increased HBP activation blunted the hypertrophic response, although relatively high *in vivo* glucose levels and downregulated seipin increased extracellular signal-regulated kinase 1/2 (ERK1/2) mediated cyclin D2 expression (cell cycle progression), as well as repressor element 1-silencing transcription factor (REST) and forkhead box proteins of class O subgroup (FOXO) *O*-GlcNAcylation, ultimately promoting hypertrophic gene expression (49, 57, 58, 101). This suggests that although the stress-hypertrophy signaling may be blunted, the HBP can promote cardiac hypertrophy independent of stress signaling. The method by which diabetes is experimentally induced, and the condition of the heart (pre-intervention) may also be influencing factors. Genetically modified rodents such as the leptin receptor-deficient (*db/db*) mice and those injected with streptozotocin display distinct pathophysiological alterations that result in diabetes but may slightly alter their susceptibility to hypertrophy (161). Moreover, the duration of high glucose treatment (*in vitro*) or pressure overload intervention (*in vivo/ex vivo*) also contributes to the degree of dysfunction observed (**Tables 2**–**4**).

In summary, increased HBP activity and *O*-GlcNAcylation is observed in the context of cardiac hypertrophy and hypertension. Increased substrate availability combined with reduced antioxidant defense systems promotes flux into the HBP and *O*-GlcNAcylation of proteins such as AMPK and CD36. However, the effects of the HBP are not entirely clear under conditions where hypertension and cardiac hypertrophy exist in combination with diabetes. The degree by which the HBP influences all three pathologies centers around the duration and intensity of the stimulus (i.e. glucose overload, oxidative stress) as well as the experimental conditions.

### Chronic HBP upregulation: Diabetic context

The greatest proportion of research work done thus far on the role of the HBP has focused on the diabetic context. In support, ~ 30% of articles cited in **Tables 1**–**4** include either glycemic conditions, contributing factors (e.g., consumption of sugar-sweetened beverages), or diabetes-related pathology within a clinical/experimental context. For example, Wang et al. (2018) investigated the effects of a hyperinsulinemic hypoglycemic clamp in type 1 diabetes mellitus patients with myocardial infarction. Here, increased *O*-GlcNAc together with lowered miRNA-24 expression levels were found in patients with attenuated cardiac function (38). Increased miRNA-24 contributes to decreased apoptosis, lowered fasting glucose and insulin levels, and attenuated autophagy-related gene 4a (ATG4a – required for autophagy), as well as attenuated *O*-GlcNAc and OGT expression, thus conferring cardioprotective effects. Therefore, increasing miRNA-24 levels in circulation may prove a beneficial therapeutic target for both diabetic and ischemic heart conditions.

Experimental studies highlighted possible mechanisms for the blunted cardiac function observed in diabetic patients (38–40). Higher circulating glucose (indicated by elevated glycated hemoglobin levels) increases insulin-independent myocardial glucose uptake (39, 54). As the Krebs cycle possesses a limited capacity to convert pyruvate into reducing equivalents, glycolytic intermediates accumulate and are shunted into NOGPs such as the HBP (as discussed previously). Increased HBP flux promotes atrial fibrillation through calcium-calmodulin-dependent protein kinase II-dependent (CaMKII – cardiomyocyte calcium homeostasis) and independent mechanisms, respectively (59, 102). Elevated *O*-GlcNAcylation of key enzymes can increase oxidative stress, impair DNA repair machinery (8-oxoguanine DNA glycosylase (Ogg1)), increase apoptotic/autophagic stimuli, and promote mitochondrial stress. The combination of such PTMs contributes towards cardiac fibrosis, hypertrophy, and increased susceptibility to ischemic injury (26–29, 39, 46, 50, 55–65, 81, 82, 100, 127, 129–132, 134). Proteomic analysis suggests a potential role of *O*-GlcNAcylation in regulating myocardial actin (serine^54^) and troponin (serine^150^) functionality (162). While this may improve function in an acute context, chronic *O*-GlcNAcylation observed in conditions such as diabetes may result in decreased submaximal force development and reduced cardiac contractility (162). Increased HBP flux is also associated with metabolic derangements, lowered ketone utilization in the presence of enhanced production and availability, and increased aldehydes and FFAs (46, 50–53). Decreasing *O*-GlcNAcylation through OGA overexpression can lower p53 (“Guardian of the Genome”) expression thus inhibiting apoptotic signaling and improving cardiac function (62).

The majority of interventions in the context of diabetes are not preventative, and instead, focus on mitigating the effects of the HBP under established diabetic conditions. An interesting discrepancy is observed when comparing the findings of swim-exercised streptozotocin diabetic mice and treadmill-exercised *db/db* mice. Exercise significantly decreased cardiac *O*-GlcNAc in streptozotocin mice through increased OGA activity and expression, despite chronic hyperglycemia (163). However, *db/db* mice subjected to treadmill-exercise displayed increased cardiac protein *O*-GlcNAcylation and an upregulated hypertrophic response (125). Exercise was also investigated in the context of cardiac hypertrophy, where a single bout was sufficient to downregulate cytosolic protein *O*-GlcNAcylation and promote adaptive hypertrophic signaling (164). The discrepancy in findings may be in part due to the type of diabetic animal model utilized as well as the intensity and duration of the exercise protocol. Alternative interventions include vitamin D which lowers flux into NOGPs such as the HBP and AGE-RAGE pathways, mitigating protein *O*-GlcNAcylation and inflammation, respectively (66).

There is extensive evidence supporting increased HBP activity under diabetic conditions, regardless of the experimental induction. Increased *O*-GlcNAcylation of various proteins predisposes the heart to pathology through several mechanisms as highlighted in **Tables 1**–**4**. Combined with increased oxidative stress potentiating greater HBP activation, such dysfunctionality predisposes the heart to impaired contractility, hypertrophy, and fibrosis. These hallmarks of heart failure observed at a high frequency in diabetic patients, provide a mechanistic link between the two pathologies.

### Heart failure: Role of the HBP

Clinical studies highlight increased *O*-GlcNAc levels in the myocardial tissue of patients with heart failure (ejection fraction < 20%) which provides a final clinical endpoint for enhanced HBP flux over an extended period (48). Moreover, reduced cardiac function under diabetic and non-diabetic conditions may be attributed to increased cardiac *O*-GlcNAcylation of target proteins (46–48). Diabetic patients diagnosed with heart failure also displayed attenuated ketone utilization, suggesting that alternative fuel substrates enter the heart, yet their potential energy is not harnessed (46).

Umapathi et al. (2021) highlighted the role of excessive *O*-GlcNAcylation in cardiac failure and sudden death (115). Transgenic mice overexpressing OGT developed significant cardiac pathologies including arrhythmias and dilation (115). Of note, crossing OGT transgenic mice with those overexpressing OGA, negated the detrimental effects of increased HBP activity. One key finding was that the *O*-GlcNAcylation of mitochondrial complex I impaired its function and activity (115). Mitochondrial complex I is considered the rate-limiting component of oxidative respiration and without sufficient activity, energy production is severely attenuated. Subsequently, cardiomyocytes are unable to contract efficiently, and the ejection fraction is ultimately reduced – thus returning to the diagnostic feature of heart failure.

A recently emerging biomarker of heart failure is miRNA-423-5p as congestive heart failure patients displayed increased plasma levels (139, 140). OGT is a downstream target with its expression significantly lowered in cardiomyocytes with increased expression of this miRNA. While this may appear beneficial in reducing *O*-GlcNAcylation, apoptotic stimuli and ubiquitin proteosome activity are elevated, thus contributing to cardiomyocyte death independently of chronic *O*-GlcNAcylation. Another diagnostic marker for heart failure is the increased serine phosphorylation of insoluble desmin (94). Desmin is a target of *O*-GlcNAcylation and is intricately involved in cardiac contractility (165, 166). Although site-specific *O*-GlcNAcylation can indirectly promote enzymatic activity by allosterically activating the phosphorylation site, this is not the case with desmin. Here, increased desmin *O*-GlcNAcylation does not alter its phosphorylation status but does confer improved cardiac function (94). These data indicate that this may not be solely attributed to desmin *O*-GlcNAcylation (no significant changes in expression) and instead likely be due to the acute upregulation of other *O*-GlcNAcylation targets during heart perfusion.

Autophagy plays a substantial role in the progression of heart failure and is also influenced by *O*-GlcNAcylation. Although pre-clinical work resulting in the downregulation of OGT expression indicates the role of *O*-GlcNAcylation in the initial phases of autophagy, chronic HBP upregulation hinders this process by modifying synaptosomal-associated protein 29 (SNAP29 – involved in the formation of the autophagolysosome) (60). Misfolded proteins and damaged organelles are subsequently unable to be degraded and interfere with intracellular homeostasis thus increasing the heart’s susceptibility to ischemic and hemodynamic injury (116). In support, therapeutic interventions aimed at increasing autophagy (e.g. spermidine) showed an improvement in cardiac health and function.

The role of *O*-GlcNAcylation in heart failure is somewhat controversial. Increased *O*-GlcNAcylation levels are observed in patients with heart failure and may contribute to autophagic dysregulation, impaired mitochondrial respiration, and altered ketone metabolism (**Tables 1**–**4**). However, diagnostic markers of heart failure (e.g. miRNA-423-5p) decrease *O*-GlcNAcylation which should provide protection; yet miRNA-423-5p promotes cardiomyocyte apoptosis independently of the HBP (139, 140). Thus, the HBP is not the sole contributor to a decreased ejection faction in the context of heart failure.

### Regulating the HBP: Benefits and drawbacks

Substrate availability, circadian rhythm, and intracellular oxidative status all govern the activity of the HBP (32). Increased glycolytic flux with limited mitochondrial oxidative phosphorylation feeds glycolytic intermediates into the HBP, which results in a plethora of downstream PTMs. As the majority of such PTMs are detrimental to cardiovascular health in a chronic setting, there are emerging interventions to mitigate such detrimental effects.

**Figure 2:**
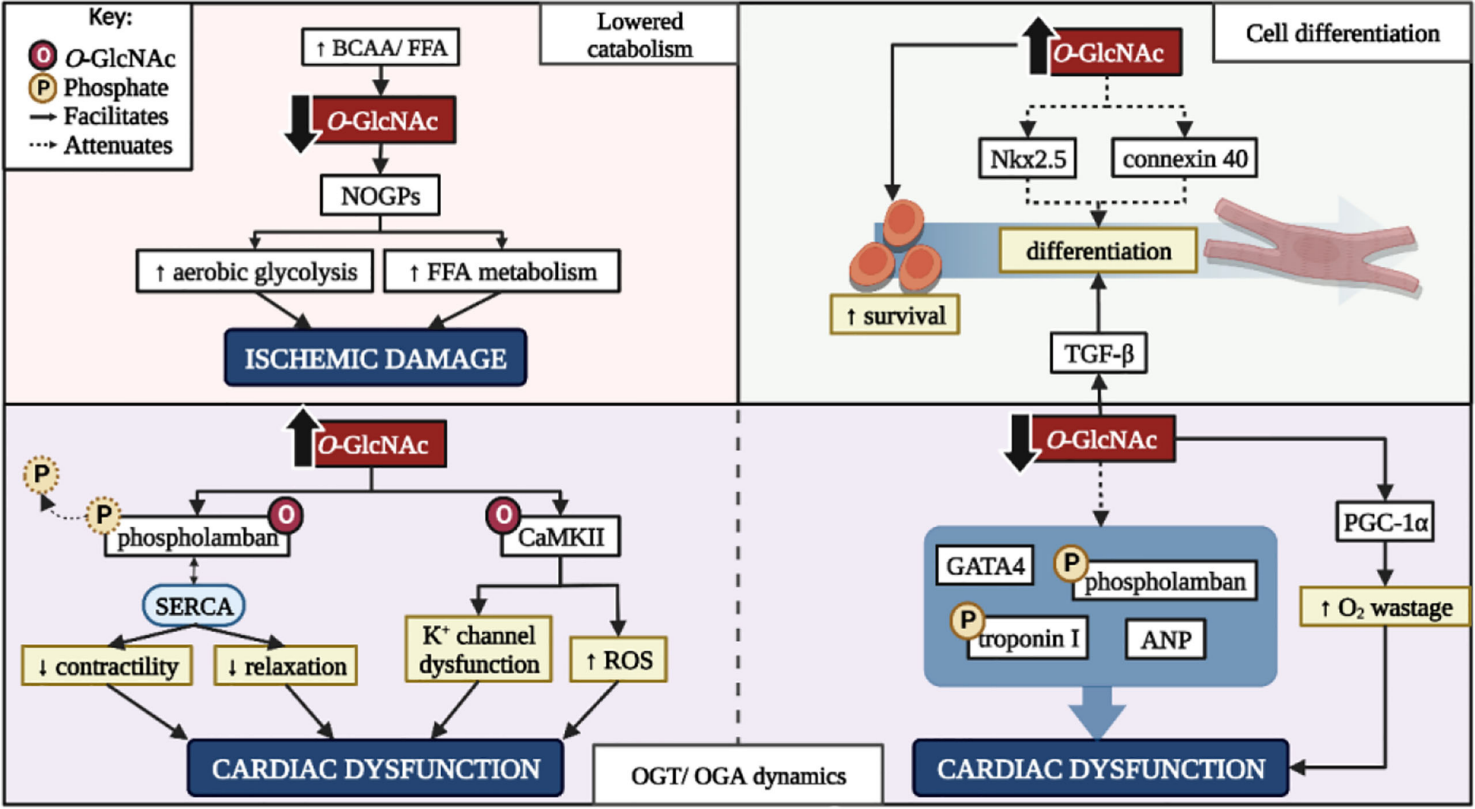
The benefits and drawbacks of altering cardiac *O*-GlcNAcylation. BCAA, branched-chain amino acid; FFA, free fatty acid; NOGPs, non-oxidative glucose pathways; K^+^, potassium; ROS, reactive oxygen species; CaMKII, calcium-calmodulin dependent protein kinase II; GATA4, GATA binding protein 4; ANP, atrial natriuretic peptide; PGC-1α, peroxisome proliferator-activated receptor-gamma coactivator-1α; OGT, *O*-GlcNAc transferase; OGA, *O*-GlcNAcase. *Created with BioRender.com*.

Dietary supplementation with BCAAs influences cardiac glucose metabolism in a ratio-specific manner regarding its preferred substrates. A low BCAA/FFA ratio and glucose metabolism (enhanced catabolism) is beneficial while higher BCAA levels (lowered catabolism) can be damaging (107). Pre-clinical data highlight the effects of BCAA catabolic alterations (107, 167). Accumulation of BCAAs (lowered catabolism) inhibits the HBP and decreases *O*-GlcNAc PTMs, lowers PDH activity and myocardial glucose uptake, and promotes heart failure under conditions of pressure overload (107, 167). Decreased *O*-GlcNAc combined with lowered PDH activity suggest that glycolytic intermediates move into alternative NOGPs such as the AGE-RAGE or polyol pathway (**Figure 2**). Due to PDH inactivation, aerobic and anaerobic glycolysis are delinked, and the heart is therefore forced to rely on FFAs for mitochondrial ATP production. This results in increased injury during anoxic conditions due to reduced glucose catabolism, while excessive FFA utilization increases susceptibility to ischemic damage upon reperfusion – further compounding ischemic damage (107, 167). Promoting glucose metabolism by upregulating glucose transporter 1 (GLUT1) receptors rescues the heart from these damaging metabolic alterations and lowers ischemic damage (107). In contrast, enhanced BCAA catabolism is beneficial under certain circumstances, for e.g. it promotes survival through improving anti-oxidant defense systems and mitochondrial biogenesis in the heart following dietary supplementation (168). Therefore, the beneficial effects of BCAAs regarding the attenuation of HBP flux depends on various factors and the intracellular milieu at the time. This suggests that the downstream implications of proposed interventions to lower HBP activity need to be weighed against their potential benefits in a context-specific manner.

The impact of the HBP on *in vitro* cell differentiation is a lesser investigated area of research. The HBP is integral in mesenchymal stromal cell differentiation which extends to adult and embryonic cardiac stem cells (169–171). Evidence supports the shift away from oxidative metabolism and towards biosynthetic pathways to provide substrates for anabolic processes (170). However excessive *O*-GlcNAcylation hinders cardiac cell differentiation and may potentiate cardiomyopathy (171). Increased HBP activity decreases Nkx2.5 and connexin 40, whilst increasing smooth muscle actin expression (169). Nkx2.5 is heavily involved in cardiomyocyte cell fate during embryonic development and mutations within this gene are associated with congenital heart defects (172). Increased *O*-GlcNAcylation inhibits Nkx2.5 promotor activation thus attenuating its expression. Connexin 40 is a gap-junction protein intricately involved in the electrical conducting system of the heart (173) and lowered expression observed under conditions of increased HBP activity may occur as a direct or indirect result of *O*-GlcNAcylation, or Nkx2.5 expression (169). The combination of decreased Nkx2.5 and connexin 40 expression due to increased *O*-GlcNAcylation in adult cardiac mesenchymal cells limits their ability to differentiate and thus lower their efficacy as a cell-based therapeutic intervention (**Figure 2**). However, increased HBP activity in cardiac stem cells augments their regenerative capabilities and enhances cellular survival post-transplant (174). This further highlights the potential benefits and drawbacks of targeting increased HBP flux.

Aside from HBP activity regulated by GFAT1/2, the dynamics between OGT and OGA also determine its influence on cardiac function. For example, an increased OGA/OGT ratio lowers overall *O*-GlcNAcylation together with increased TGF-β and peroxisome proliferator-activated receptor-gamma coactivator-1α (PGC-1α) expression and activity, and decreased GATA binding protein 4 (GATA4), atrial natriuretic peptide (ANP), p-phospholamban, and p-cardiac troponin I levels (48, 110, 111). Increased TGF-β and PGC-1α expression and activity promote myoblast differentiation and mitochondrial biogenesis, respectively (111). While this appears beneficial, increased PGC-1α activity also enhances the heart’s reliance on FFAs for energy production, thus potentiating oxygen wastage, reactive oxygen species production, and NOGP flux. Decreased GATA4, ANP, p-phospholamban, and p-cardiac troponin I expression collectively promote cardiovascular inotropy and lusitropy, fibrosis, and hypertrophy (110). Thus, the combination of events following targeted OGT inactivation or OGA hyperactivation may promote more damage than relief, depending on the context of the intervention (**Figure 2**). This targeted therapeutic approach would be beneficial to the heart in the event of established HBP overactivation, and not as a preventative or preemptive measure.

Phospholamban plays an integral role in cardiac contractility through its interactions with the sarcoplasmic/endoplasmic reticulum Ca^2+^ ATPase (SERCA) (175). Phosphorylation of phospholamban on serine^16^ renders it inactive and alleviates any inhibitory effects on SERCA, thus allowing for increased cardiac contractility and relaxation (175). However, *O*-GlcNAc targets the serine residues that alter phospholamban’s function, for e.g. Yokoe et al. (2010) demonstrated an increase in *O*-GlcNAcylated phospholamban with a concurrent decrease in phosphorylated phospholamban and cardiac function (122). Additionally, interactions of SERCA and phospholamban were increased in PUGNAc treated cells, suggesting that increased phospholamban *O*-GlcNAcylation promotes its negative regulation on SERCA. However, OGT^KO^ models indicate reduced phosphorylated phospholamban with no effect of SERCA calcium handling (110). Further studies are required to understand this dynamic relationship of phospholamban *O*-GlcNAcylation and phosphorylation. CaMKII is another protein heavily involved in cardiac contractility and the addition of *O*-GlcNAc moieties regulate its activity in a residue-specific manner. For example, serine^280^ *O*-GlcNAcylation is not required for activation yet influences potassium channel activity, while *O*-GlcNAcylation of serine^279^ autonomously activates CaMKII (57, 58, 125). Increased *O*-GlcNAcylation of CaMKII thus augments its activity and facilitates numerous downstream effects ranging from altered ion channel function and inflammatory gene transcription, culminating in the development of cardiac arrhythmias and reduced heart function (47, 127, 176).

While an increased OGA/OGT activity ratio does influence cardiac *O*-GlcNAcylation, the localization of OGA and OGT also impacts cardiac function. Under normal conditions, OGT is primarily localized to the Z-line of myofilaments with OGA to the A-band (61). The delocalization of such enzymes correlates to increased cardiac *O*-GlcNAc levels and an impaired calcium response (61). This suggests that OGT and OGA localization, as well as their activity and expression, influence their respective roles in cardiac dysfunction. Without a sufficient response to physiological calcium levels, the heart struggles to meet the required ejection fraction for normal bodily function. Compensatory mechanisms to increase cardiac output include an increase in left ventricular wall mass which contributes to cardiac hypertrophy. Therefore, the potential effects of targeted therapies to lower *O*-GlcNAcylation need to be weighed against the HBP’s physiological role.

## Conclusion

**Figure 3:**
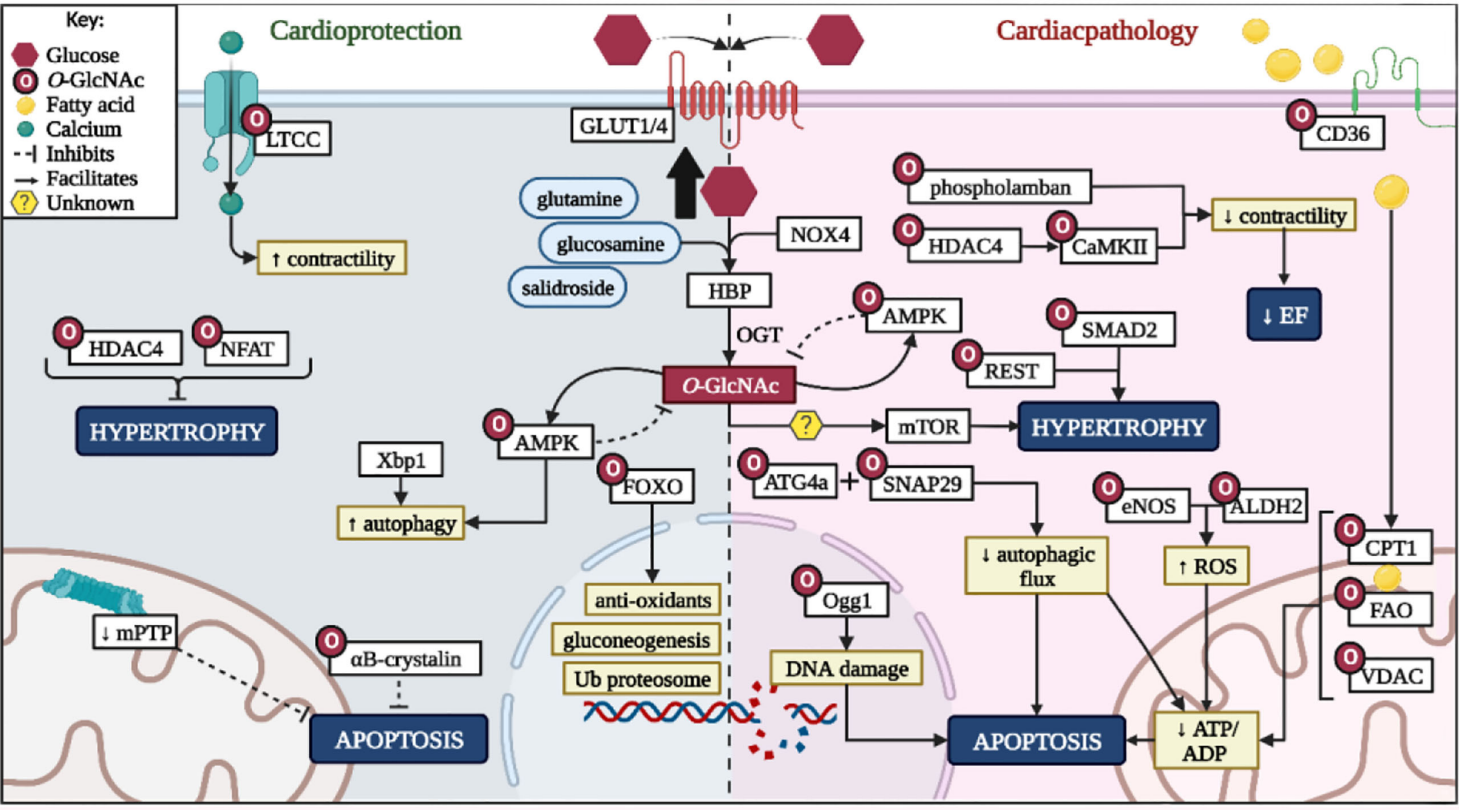
Summary of HBP role on cardioprotection and pathological effects. LTCC, L-type calcium channel; HDAC4, histone deacetylase 4; NFAT - nuclear factor of activated T-cells; Xbp1, X-box binding protein 1; GLUT1/4, glucose transporter 1/4; AMPK, 5ʹ-adenosine monophosphate-activated kinase; FOXO - forkhead box transcription factors; HBP, hexosamine biosynthetic pathway; OGT, *O*-GlcNAc transferase; NOX4, NADPH oxidase 4; ATG4a, autophagy related 4A cysteine peptidase; Ogg1 - 8-Oxoguanine glycosylase; SNAP29, synaptosomal-associated protein 29; mTOR, mammalian target of rapamycin; DNA, deoxyribonucleic acid; REST, repressor element 1-silencing transcription factor; SMAD2, mothers against decapentaplegic homolog 2; CaMKII, calcium-calmodulin dependent protein kinase II eNOS, endothelial nitric oxide; ALDH2, aldehyde dehydrogenase 2; ROS, reactive oxygen species; EF, ejection fraction; CD36, cluster of differentiation 36; CPT1, carnitine palmitoyltransferase-1/2; FAO, fatty acid β-oxidation enzymes; VDAC, voltage-dependent anion channels; ATP, adenosine triphosphate; ADP, adenosine diphosphate. *Created with BioRender.com*.

The HBP can be considered a double-edged sword in the context of the heart (**Figure 3**). Various proteins require *O*-GlcNAc moieties for activation or repression and can confer cardioprotection against hypertrophy and apoptosis. However, consistent HBP upregulation either due to intrinsic factors (i.e. circadian rhythm), substrate availability, or elevated intracellular oxidative status, contribute towards chronic protein *O*-GlcNAcylation and subsequent cardiac dysfunction (**Figure 3**). Clinical, *in vivo*, and *in vitro* studies highlighted excessive HBP activity in a number of cardiac pathologies ranging from diabetic cardiomyopathy to hypertrophy, hypertension, and heart failure (**Figure 3**). Thus, the HBP has a emerged as a promising therapeutic target, although the extent of such downregulation needs to be carefully considered as basal levels are required for optimal cardiac function.

## Author contributions

MC conceived the idea and wrote the first draft, edited and approved the final draft. DJ conceived the idea and wrote the first draft, edited and approved the final draft ME conceived the idea and wrote the first draft, edited and approved the final draft. All authors contributed to the article and approved the submitted version.

## Funding

This work is based on the research supported wholly/in part by the National Research Foundation of South Africa (MND210428597749), National Research Foundation (129325 to MFE) and National Research Foundation (MND210428597749 to MC).

## Conflict of interest

The authors declare that the research was conducted in the absence of any commercial or financial relationships that could be construed as a potential conflict of interest.

## Publisher’s note

All claims expressed in this article are solely those of the authors and do not necessarily represent those of their affiliated organizations, or those of the publisher, the editors and the reviewers. Any product that may be evaluated in this article, or claim that may be made by its manufacturer, is not guaranteed or endorsed by the publisher.
